# Moderating Effects of Parental Characteristics on the Effectiveness of a Theory of Mind Training for Children with Autism: A Randomized Controlled Trial

**DOI:** 10.1007/s10803-017-3117-1

**Published:** 2017-04-08

**Authors:** Danielle M. J. de Veld, Patricia Howlin, Elske Hoddenbach, Fleur Mulder, Imke Wolf, Hans M. Koot, Ramón Lindauer, Sander Begeer

**Affiliations:** 1 Department of Clinical Neuro and Developmental Psychology & EMGO Institute for Health and Care Research, Vrije Universiteit, Van der Boechorststraat 1, 1081BT Amsterdam, The Netherlands; 2 Institute of Psychiatry, King’s College, 16 De Crespigny Park, London, SE5 8AF UK; 3 Faculty of Health Sciences & Brain and Mind Centre, The University of Sydney, 100 Mallet Street, Camperdown, NSW 2050 Australia; 4 De Bascule, Rijksstraatweg 145, 1115AP Duivendrecht, The Netherlands; 5 Department of Child and Adolescent Psychiatry, Academic Medical Center, Meibergdreef 5, 1105 AZ Amsterdam, The Netherlands; 6 Collaborative Antwerp Psychiatric Research Institute (CAPRI), University Antwerp, Universiteitsplein 1, 2610 Wilrijk, Antwerp Belgium

**Keywords:** Autism, Treatment, Randomized controlled trial, Theory of mind, Moderator

## Abstract

This RCT investigated whether the effect of a Theory of Mind (ToM) intervention for children with ASD was moderated by parental education level and employment, family structure, and parental ASD. Children with autism aged 8–13 years (*n* = 136) were randomized over a waitlist control or treatment condition. At posttest, children in the treatment condition had more ToM knowledge, showed fewer autistic features, and more ToM-related behavior than children in the control condition. Children who had one or two parents with at least a college degree, and children with parents not diagnosed with/suspected of having ASD themselves benefitted from the training. These findings provide valuable information about family variables that need to be taken into account in treatment design and implementation.

## Introduction

Randomized clinical trials are considered the gold standard for investigating the effectiveness of psychological interventions. However, beyond establishing that a treatment is generally effective, it is important to determine for whom, and under what circumstances, intervention is most effective, in other words: what variables moderate treatment success (Kraemer et al. 2002). Although this information is particularly crucial in trials involving a condition as heterogeneous as autism spectrum disorder (ASD), there has been relatively little systematic study of child or family factors that are predictive of response in autism treatment trials. The current study aimed to investigate parent characteristics that may moderate the effectiveness of one of the most common types of intervention for school-aged children with ASD: social cognition training (Wierda et al. 2015).

Parents play an essential role in children’s development and their influence may be even more important in shaping the development of children with ASD (Prizant et al. 2003). Problems with generalization of skills in autism are well documented (cf. Plaisted 2001) and while children with ASD can learn new skills during treatment, they typically have difficulties generalizing these skills to other situations (e.g. Begeer et al. 2011). Parents can help their children practice newly acquired skills in many different, real life settings (Koegel and Kern Koegel 2006) and hence their involvement in intervention may be crucial for wider generalization and maintenance of treatment effects. The extent of parental involvement in autism treatment programs varies (Burrell and Borrego 2012) from active engagement as therapist or co-therapist (up to 7 h per week; McConachie and Diggle 2007) to a less intensive role of observer (e.g. 15 min per week; Begeer et al. 2015); parental participation also depends on whether a specific treatment is parent-mediated (Nevill et al. 2016) or directly child focused (e.g. Begeer et al. 2011). Nevertheless, despite the potential importance of parent-involvement in treatments for children with ASD, very little is known about how parental characteristics affect treatment success (Karst and Van Hecke 2012).

Although various parental factors may potentially influence treatment outcome, evidence for their impact is often conflicting. Thus, it has been suggested that parents with higher education levels may be better able to understand and implement treatment techniques (Burrell and Borrego 2012), thereby promoting learning and generalization of new skills in their child. In studies of neurotypical children, for example, higher parental education is positively associated with parents’ achievement beliefs, higher economic status, and a more stimulating home environment, all of which may increase children’s learning opportunities and attainments (Davis-Kean 2005). In the field of autism, Ben Itzchak and Zachor (2011) found that higher maternal education level was associated with greater gains in child cognitive abilities following either an Applied Behavior Analysis or eclectic center-based intervention. However, Magiati et al. (2007) found no relationship between parental education level and children’s progress after 2 years of Early Intensive Behavioral Interventions or autism specific nursery provision. Both of these studies involved young children and, to our knowledge, there are currently no studies on the moderating role of parental education in treatments for older children with ASD.

Parental employment and/or financial status may also be associated with better treatment outcomes since being in paid employment may decrease financial and other strains on families. Evidence for this suggestion is partly based on other clinical groups, e.g., obese children (Röbl et al. 2013) and children with disruptive behavior disorders (Shelleby and Kolko 2015). In ASD, parent-reported financial strain was correlated with poorer treatment outcomes in a sample of young children with ASD (Gabriels et al. 2001). However, income did not predict the effectiveness of a parent-training program to reduce noncompliance in children with ASD and disruptive behavior (Farmer et al. 2012). Thus, the specific relation between parental employment status and treatment outcome in children with ASD remains uncertain.

The wider family structure is another factor potentially influencing treatment effectiveness. Thus, households consisting of two biological or adoptive parents tend to report less parental stress than single mothers or families with at least one step-parent (NSCH 2011/2012). In line with this, children with autism from intact, two-parent households were found to gain most from a family-oriented treatment program (compared to children from single or divorced parents; Robbins et al. 1991). Also, high levels of parenting stress limited the effects of early teaching interventions and pivotal response training for children with ASD (Osborne et al. 2008; Robbins et al. 1991).

A further factor, about which surprisingly little is known, is the impact of parental levels of autistic traits. If parents themselves have features of the broader autism phenotype (BAP; Parr et al. 2015a) this might enable them to identify more easily with their child’s difficulties. On the other hand, if parents, too, have autistic-type limitations in flexibility and learning, this could make it more difficult for them to help their child acquire and generalize new skills (Karst and Van Hecke 2012; Parr et al. 2015b). For example, parents with BAP traits may have difficulties in recognizing situations that allow the child to practice newly taught skills. BAP characteristics may also influence parents’ perspective on how feasible, and/or useful the treatment is for their child (Gerdts and Bernier 2011), thereby affecting their motivation to co-operate in therapy.

In this study, we investigated whether these particular characteristics, i.e., parental education, employment, family structure, level of autism traits, moderated the effectiveness of a social cognition intervention (Theory of Mind (ToM) training) for 8-to-13-year-old children with ASD. Previous data from this randomized control trial (RCT) showed that training improved children’s knowledge of ToM; ToM-related behavior also increased and autistic features decreased (Begeer et al. 2015). A larger sample of participants from the same RCT has since become available, allowing us to test the potential moderating effects of these parental characteristics. Specifically, we hypothesized that the ToM training would be more effective for children:

I. With parents of higher education levels,
II. With parents in paid employment,
III. Growing up in a family structure with two biological parents.

The analyses regarding parental ASD were of an exploratory nature, as a lack of research in this area limits specific hypotheses.

## Method

### Design

The study was a randomized controlled trial with a waitlist control group and an intervention group. The project was approved by the Medical Ethics Committee of the VU University Medical Center (Project No. 2010/241). The trial protocol was registered at the Netherlands Trial Register (http://www.trialregister.nl, Trial No. 2327) before it started and published prior to completion of the data collection (Hoddenbach et al. 2012).

### Participants

**Fig. 1:**
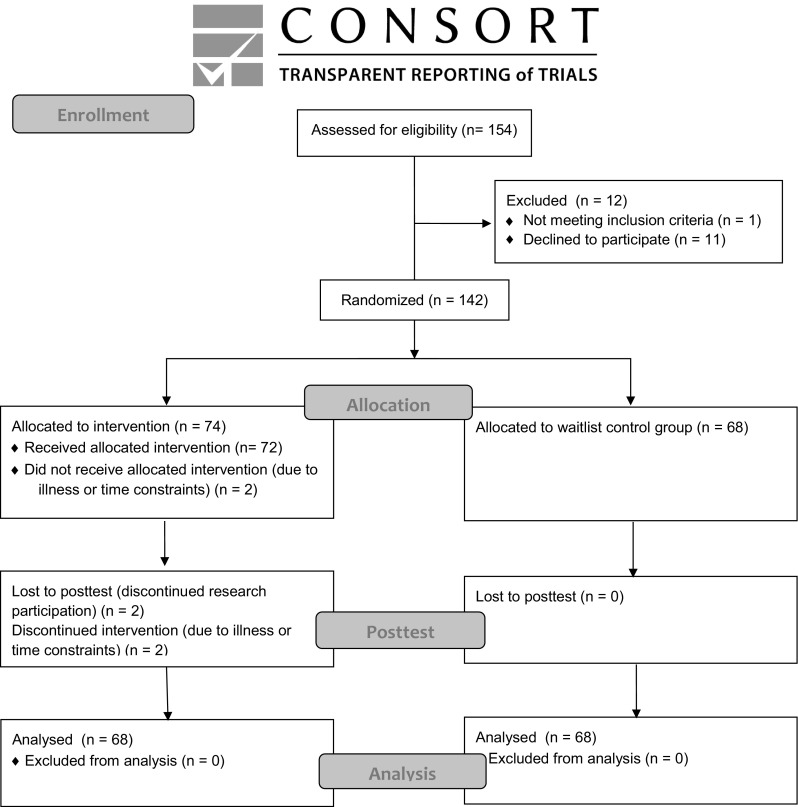
CONSORT 2010 flow diagram of participant flow through the study

The sample comprised 136 children (90% boys) aged between 8 and 13 years (*M* = 9.66, SD = 1.23) meeting the eligibility criteria of: (1) an ASD according to the DSM-IV-TR (APA 2001), based on multiple assessments by psychologists and psychiatrists not involved in this study; (2) a verbal IQ score >70 based on the Peabody Picture Vocabulary Test-III-NL (PPVT) (Dunn and Dunn 1997). Parents gave informed consent prior to study participation. Figure 1 shows participant flow through the study. Characteristics of the sample are summarized in Table 1. The mean number of previously received treatments was 1.01 (SD = 1.56); 34.1% of children were using medication.

**Table 1:** Baseline demographic and clinical characteristics of the ToM treatment and the waitlist control groups

	ToM treatment total n = 68^a^		Waitlist control total n = 68^a^		Test (t-test, Mann–Whitney U, or χ^2^)
	n	M (SD)	n	M (SD)	
Child gender					
Male	61		61		*χ* ^*2*^(1) = 0.00, *p* = 1.00
Female	7		7		
Child age	66	9.76 (1.30)	67	9.57 (1.17)	*t*(131) = − 0.87, *p* = 0.39
Verbal ability	66	106.95 (13.33)	67	106.19 (12.03)	*t*(131) = − 0.35, *p* = 0.73
SRS pretest	66	84.45 (20.31)	64	81.46 (21.63)	*t*(132) = 0.83, *p* = 0.41
Number of parents with a college degree					*U* = 1578.5, *z* = −1.24, *p* = 0.22
0	22		26		
1	14		20		
2	23		15		
Number of parents in paid employment					*U* = 2037.5, *z* = −0.06, *p* = 0.95
0	1		4		
1	19		16		
2	43		45		
Family structure					*χ* ^*2*^(2) = 3.68, *p* = 0.16
2 biological parents	48		40		
2 parents (coparenting or 1 stepparent)	9		10		
Single parent	7		15		
One or both parents diagnosed with/suspected of having an ASD	61		66		*χ* ^*2*^(1) = 0.31, *p* = 0.58
Yes	14		18		
No	47		48		

*SRS* Social Responsiveness Scale

^a^Some data missing for some participants

### Procedure

Participants were recruited from De Bascule, an academic center for child and adolescent psychiatry in Amsterdam, Netherlands, between April 2010 and September 2015. An independent researcher randomized the participating children to the treatment or wait list control condition using a digital random number generator. The randomization outcome was shared with the study coordinator, who informed patients about allocation outcome. In the treatment group, pre-trial assessment took place immediately prior to intervention, and post-trial assessment was conducted immediately post intervention (baseline to post intervention = 8 weeks). The waitlist control group was assessed 8 weeks prior to intervention and re-assessed immediately prior to intervention (baseline to post intervention = 8 weeks). More detailed information on the procedure is available in the published trial protocol at http://www.trialsjournal.com (Hoddenbach et al. 2012).

### Intervention

The “Mini ToM intervention” is a manualized, weekly intervention comprising eight 1-h sessions, provided to five to six children at a time, all aged within 3 years of each other. The training is delivered in a child psychiatric center by certified therapists (licensed Counseling Psychologists, M.Sc. or Ph.D., registered with the Mental Health Council) who were trained to administer the therapy. The program is based on a validated ToM intervention (Begeer et al. 2011; Steerneman et al. 1996), and was shortened to be more cost-effective whilst retaining the key elements of the training and maintaining its effectiveness (Begeer et al. 2015). All sessions followed the same structure: (1) discussing the homework assignment; (2) games and exercises related to the day’s theme (e.g. perspective taking, emotion understanding); (3) children summarizing the session to their parents; and (4) explanation of next week’s homework assignment (including e.g. drawing an object from different angles, observing emotions in everyday life). Parents were involved in the training through two 1-h parent-sessions that explained theory of mind, the ToM-training, and how parents could help their children acquire these new skills and promote generalization. More detailed information on the treatment is available in the published trial protocol at http://www.trialsjournal.com (Hoddenbach et al. 2012).

### Descriptive Measures

#### Peabody Picture Vocabulary Test-III-NL (PPVT)

The PPVT (Dunn and Dunn 2004) was used to assess children’s verbal ability. The PPVT provides a standardized score and verbal IQ equivalent, and correlates highly with the WISC-III verbal IQ (Hodapp and Gerken 1999). Internal consistency is high (α between 0.92 and 0.98; split-half reliability between 0.86 and 0.97), as is test-reliability (r between 0.91 and 0.94; Dunn and Dunn 2004).

### Outcome Measures

#### Proximal Primary Outcome Measure: ToM Test

The ToM test (Muris et al. 1999) was chosen to assess children’s theory of mind knowledge. It comprises a standardized, 72-item, interview for children aged 5–13 years, and measures ToM knowledge at three levels (Elementary, Intermediate, and Complex), with cognitive sub-stages within each level (perception and imitation, emotion recognition, elementary theory of mind, second-order belief understanding, and understanding of complex humor). Children are asked to look at a picture and/or listen to a story and answer the corresponding question. Items are scored 0 (incorrect) or 1 (correct); a higher total score indicates greater ToM knowledge. Internal consistency of the task ranges from 0.80 to 0.92; concurrent validity with traditional ToM tasks is high (r between 0.37 and 0.77), and test–retest reliability is satisfactory (ICC between 0.80 and 0.99; Muris et al. 1999).

#### Distal Primary Outcome Measure: ToM Behavior Checklist (ToMbc)

The ToMbc (Begeer et al. 2015) was chosen to assess ToM-related behavior in everyday life. On this 8-item questionnaire parents indicate the frequency, over the last week, of specific ToM-related behaviors of their child across eight domains of behavior (understood a joke, comforted somebody, asked about someone’s feelings, figured out his/her story was not interesting to others, apologized, paid close attention to somebody’s story, spontaneously complimented someone, asked an interested question). Frequency of occurrence of each domain is rated from 0 (never) to 5 (very often). A higher total score indicates a higher frequency of ToM-related behaviors. Reliability has been found to be good (α = 0.81; Begeer et al. 2015).

#### Distal secondary Outcome Measure: Social Responsiveness Scale (SRS)

The SRS (Constantino and Gruber 2007) was chosen to assess autistic features. It is a 65-item parent questionnaire, divided into five subscales: social awareness, social cognition, social communication, social motivation, and autistic mannerisms. Parents rate each item from 0 (never true) to 3 (almost always true) and a higher total score indicates more autistic features. Internal consistency (0.91–0.97), test–retest reliability (0.84–0.97), interrater reliability (0.76 and 0.95) are good (Bolte et al. 2008).

### Moderators (Parental Education, Parental Employment, Family Structure, and Parental ASD)

At pretest, parents completed a questionnaire regarding several sociodemographic characteristics.

#### Parental Education

Parental education was assessed on a scale ranging from 1-primary school to 7-university education for both parents separately. For use in the current study this information was recoded to represent the number of parents (0–2) with at least a college degree.

#### Parental Employment

Parents were asked to indicate whether they were in paid employment. The number of parents in paid employment (0–2) was used in subsequent analyses.

#### Family Structure

Parents indicated the family structure in which their child was currently growing up. For the present analysis, three categories were distinguished: two biological parents; two parents but consisting of either separated biological parents (co-parenting) or one biological parent and one step parent; and single parent.

#### Parental ASD

Parents were asked to indicate if any family members, other than the participating child, were either diagnosed with, or suspected of having, an ASD. Due to the small number of cases in which both parents were diagnosed with/suspected of having an ASD, this variable was recoded to a dummy variable indicating whether the child did (1) or did not (0) have at least one parent with a diagnosis or suspicion of an ASD.

### Statistical Analyses

Data were analyzed using hierarchical multiple linear regression analyses. The first step included pretest values on the respective dependent variable, and the main effects for condition and the moderator under investigation. The second step added the condition*moderator interaction. Continuous moderator variables were centered by subtracting their means. Categorical moderators were investigated using dummy coding. Condition was coded as: 0 = control; 1 = treatment. Level of significance was set at *p* < 0.05.

## Results

Table 2 shows the results of all the regression analyses. The main results are outlined below.

**Table 2:** Results of hierarchical multiple regression analyses predicting posttest scores on the different outcome measures

Predictor Step	ToM test			ToMbc			SRS		
	b (SE)	Part 2	${\text{R}}_{{\text{change}}}^2$	b (SE)	Part 2	${\text{R}}_{{\text{change}}}^2$	b (SE)	Part 2	${\text{R}}_{{\text{change}}}^2$
Parental education									
Step 1			0.54***			0.52***			0.65***
Pretest score	0.56 (0.06)	0.32***		0.60 (0.07)	0.36***		0.77 (0.06)	0.56***	
Verbal IQ	0.07 (0.03)	0.02*		0.01 (0.03)	0.00		0.14 (0.10)	0.01	
Condition	3.97 (0.76)	0.12***		2.15 (0.68)	0.05**		−7.97 (2.50)	0.03**	
Parental education	−0.72 (0.48)	0.01		0.02 (0.41)	0.00		−1.85 (1.52)	0.00	
Step 2			0.03**			0.02^+^			0.01^+^
Pretest score	0.57 (0.06)	0.33***		0.59 (0.07)	0.35***		0.77 (0.06)	0.55***	
Verbal IQ	0.08 (0.03)	0.03**		0.00 (0.03)	0.00		0.16 (0.10)	0.01	
Condition	4.13 (0.74)	0.13***		2.17 (0.67)	0.05**		−7.96 (2.46)	0.03**	
Parental education	−2.14 (0.68)	0.04**		0.77 (0.57)	0.01		−4.73 (2.15)	0.02*	
Condition × parental education	2.53 (0.89)	0.03**		−1.45 (0.78)	0.02^+^		5.49 (2.92)	0.01^+^	
Parental employment									
Step 1			0.51***			0.54***			0.66***
Pretest score	0.56 (0.06)	0.36***		0.64 (0.06)	0.43***		0.82 (0.06)	0.61***	
Condition	4.00 (0.74)	0.12***		1.65 (0.64)	0.03*		−7.48 (2.38)	0.03**	
Parental employment	0.40 (0.67)	0.00		0.97 (0.57)	0.01^+^		0.66 (2.15)	0.00	
Step 2			0.00			0.01			0.00
Pretest score	0.56 (0.06)	0.36***		0.65 (0.06)	0.43***		0.82 (0.06)	0.61***	
Condition	4.00 (0.74)	0.12***		1.64 (0.64)	0.03*		−7.52 (2.38)	0.03**	
Parental education	0.93 (0.86)	0.00		1.48 (0.73)	0.02*		−0.37 (2.74)	0.00	
Condition × parental employment	−1.31 (1.35)	0.00		−1.32 (1.20)	0.00		2.69 (4.44)	0.00	
Family structure									
Step 1			0.51***			0.53***			0.66***
Pretest score	0.56 (0.06)	0.36***		0.64 (0.06)	0.44***		0.80 (0.06)	0.58***	
Condition	3.90 (0.75)	0.11***		1.85 (0.65)	0.03**		−7.28 (2.40)	0.03**	
Single parent	− 0.40 (1.02)	0.00		1.24 (0.91)	0.01		2.68 (3.39)	0.00	
Coparent/stepparent	0.26 (1.09)	0.00		−0.06 (0.90)	0.00		0.62 (3.38)	0.00	
Step 2			0.00			0.01			0.00
Pretest score	0.56 (0.06)	0.35***		0.62 (0.06)	0.39***		0.80 (0.06)	0.58***	
Condition	4.09 (0.91)	0.08***		2.06 (0.77)	0.03**		−8.14 (2.85)	0.02**	
Single parent	0.20 (1.29)	0.00		2.00 (1.11)	0.01^+^		1.85 (4.07)	0.00	
Coparent/stepparent	0.01 (1.53)	0.00		− 0.48 (1.22)	0.00		−1.11 (4.62)	0.00	
Condition × single parent	−1.67 (2.13)	0.00		−2.58 (2.02)	0.01		2.30 (7.41)	0.00	
Condition × coparent/stepparent	0.56 (2.20)	0.00		1.00 (1.80)	0.00		3.73 (6.82)	0.00	
Parental ASD									
Step 1			0.49***			0.54***			0.65***
Pretest score	0.56 (0.06)	0.36***		0.66 (0.06)	0.44***		0.81 (0.06)	0.57***	
Condition	3.76 (0.77)	0.11**		1.62 (0.66)	0.02**		−6.97 (2.41)	0.03**	
Parental ASD	−0.29 (0.90)	0.00		1.10 (0.74)	0.1		1.40 (2.85)	0.00	
Step 2			0.02*			0.00			0.00
Pretest score	0.57 (0.06)	0.37***		0.66 (0.06)	0.44***		0.81 (0.06)	0.56***	
Condition	4.70 (0.86)	0.13***		1.36 (0.76)	0.01*		−7.04 (2.80)	0.02**	
Parental ASD	1.52 (1.20)	0.01		0.65 (1.01)	0.00		1.30 (3.73)	0.00	
Condition × Parental ASD	−3.93 (1.77)	0.02*		1.00 (1.50)	0.00		0.25 (5.61)	0.00	

^+^ *p* < 0.1; *p < 0.05; **p < 0.01; ***p < 0.001

### Parental Education

Because the nonparametric correlation between the child’s verbal IQ and the number of parents with at least a college degree was significant (*r*_*s*_ = 0.24, *p* = 0.01), the child’s verbal IQ was included in the first step of all regression models.

**Fig. 2:**
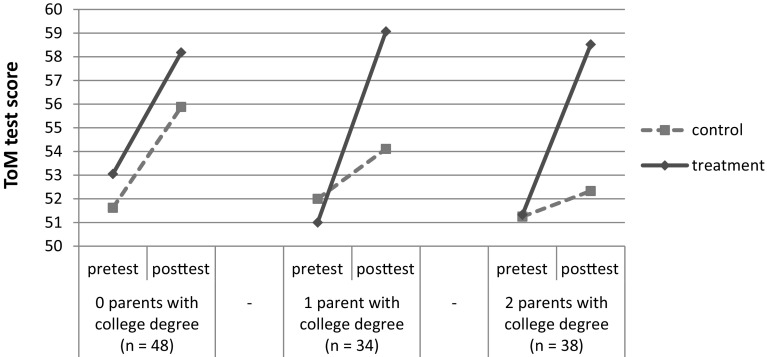
Pretest and posttest scores on the ToM test according to participant’s number of parents with at least a college degree

Step 1 models were all significant (ToM test: *F*_(4,108)_ = 31.05, *p* < 0.001, *R* ^2^ = 0.54; ToMbc: *F*_(4,105)_ = 27.82, *p* < 0.001, *R* ^2^ = 0.52; SRS: *F*_(4,105)_ = 48.55, *p* < 0.001, *R* ^2^ = 0.65). Children in the treatment condition showed better ToM knowledge (*β* = 0.35, *p* < 0.001), more ToM-related behaviors (*β* = 0.22, *p* < 0.01), and fewer autistic features (*β* = −0.19, *p* < 0.01) at posttest than those in the control condition. Higher child verbal IQ was associated only with greater ToM knowledge (*β* = 0.17, *p* < 0.05). There were no main effects of parental education. However, adding the interaction term in Step 2 significantly improved the model for the ToM test only (*F*_*change*(1,107)_ = 8.13, *p* = 0.005, $R_{{\text{change}}}^2=0.03$). Figure 2 indicates that the treatment effect became more pronounced as the number of college educated parents increased (i.e. the children in the control group showed a smaller increase in ToM knowledge from pre- to post-test with every increase in the number of college-educated parents; *β* = 0.27, *p* < 0.01).

### Parental Employment

All models for parental employment were significant at Step 1 (ToM test: *F*_(3,117)_ = 40.99, *p* < 0.001, *R* ^2^ = 0.51; ToMbc: *F*_(3,116)_ = 44.51, *p* < 0.001, *R* ^2^ = 0.54; SRS: *F*_(3,117)_ = 73.89, *p* < 0.001, *R* ^2^ = 0.66). Again, children in the treatment condition showed better ToM knowledge (*β* = 0.35, *p* < 0.001), more ToM-related behaviors (*β* = 0.17, *p* < 0.05), and fewer autistic features (*β* = −0.17, *p* < 0.01) than children in the control condition. However, there were no main effects of parental employment and adding the interaction terms did not improve the models.

### Family Structure

Step 1 models for family structure were all significant (ToM test: *F*_(4,117)_ = 29.95, *p* < 0.001, *R* ^2^ = 0.51; ToMbc: *F*_(4,115)_ = 32.85, *p* < 0.001, *R* ^2^ = 0.53; SRS: *F*_(4,116)_ = 55.31, *p* < 0.001, *R* ^2^ = 0.66). As in the previous analysis, children in the treatment condition showed better ToM knowledge (*β* = 0.34, *p* < 0.001), more ToM-related behaviors (*β* = 0.19, *p* < 0.01), and fewer autistic features (*β* = −0.17, *p* < 0.01) than children in the control condition. There were no main effects of family structure, and adding the interaction terms did not improve the models.

### Parental ASD

**Fig. 3:**
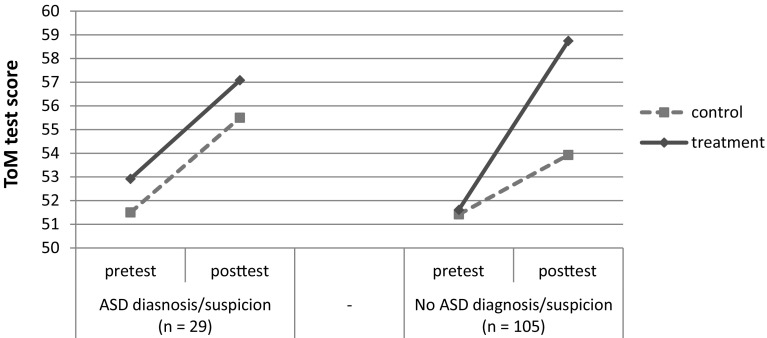
Pretest and posttest scores on the ToM test according to participant’s parental ASD status

All models for parental ASD were significant at Step 1 (ToM test: *F*_(3,116)_ = 36.58, *p* < 0.001, *R* ^2^ = 0.49; ToMbc: *F*_(3,114)_ = 45.06, *p* < 0.001, *R* ^2^ = 0.54; SRS: *F*_(3,115)_ = 71.20, *p* < 0.001, *R* ^2^ = 0.65). Once again, children in the treatment condition showed better ToM knowledge (*β* = 0.33, *p* < 0.001), more ToM-related behaviors (*β* = 0.19, *p* < 0.01), and fewer autistic features (*β* = −0.16, *p* < 0.01) than children in the control condition. There were no main effects of parental ASD, but adding the interaction term in Step 2 significantly improved the model for the ToM test only (*F*_*change*(1,115)_ = 4.96, *p* = 0.03, ${\text{R}}_{{\text{change}}}^2=0.02$). For children without a parent diagnosed with/suspected of having ASD, there was a significant treatment effect: children in the treatment condition showed better ToM knowledge than those in the control condition (*β* = −0.21, *p* < 0.05). However, for children with at least one parent with an ASD diagnosis/suspicion there was no significant treatment effect (*β* = 0.07, *p* = 0.62; see Fig. 3).

## Discussion

This study investigated whether parent characteristics moderated the effectiveness of a ToM focused training for children with ASD. Overall, the training was effective in increasing ToM knowledge, increasing ToM-related behaviors, and reducing autistic features. Examination of family factors indicated no significant effects of parental employment or family structure on any outcome measures. The effects of parental education were mixed, in that there was no association with outcome in the treatment group but children in the control group showed smaller increases in ToM knowledge as the number of parents with a college education increased. Parental ASD negatively influenced treatment effects on ToM knowledge but there were no effects on ToM-related behaviors or child autistic features.

We had predicted an effect of parental education because more highly educated parents may be better able to understand and implement treatment techniques (Burrell and Borrego 2012). Contrary to our hypothesis, in the treatment condition, children with more highly educated parents performed no better than children of less educated parents. However, in the control condition, higher parental education was associated with poorer child ToM knowledge. The reasons for this are unclear but it is possible that for control children without college-educated parents, the mere administration of the ToM test provided a new learning opportunity, thereby leading to a greater increase in ToM knowledge among this group. This finding highlights the need for more individually tailored interventions that take into account factors such as existing knowledge and skills, in both children and their parents.

The effect of the ToM training on ToM knowledge was only significant for children whose parents were not diagnosed with or suspected of having an ASD. This is consistent with the suggestion that parents who are on the spectrum themselves may have more difficulty helping their children acquire and generalize new skills in treatment (Karst and Van Hecke 2012; Parr et al. 2015b). These parents may benefit from additional guidance or coaching during the treatment. Alternatively, their children might benefit from the involvement of another closely involved adult, such as a teacher or non-BAP grandparent.

Although increases in child ToM knowledge, the primary outcome measure most closely related to the focus of the intervention, were affected by parental education and parental ASD, these factors did not affect more distal outcome measures, notably parental reports of child autistic features and ToM-related behavior. While treatment did improve children’s functioning in these domains, effect sizes were smaller than for the more proximal outcome measure. There are many unexplored variables, such as other family factors, school issues, and social relations (de Rosnay et al. 2014) that may have greater impact on behavioral, rather than knowledge outcomes. For example, social interactions with siblings, classmates or peers provide more opportunities for the display of ToM related behaviors or autistic features but the study design did not permit investigation of behavioral change in these settings.

Contrary to our expectations, parental employment did not moderate any treatment effects. However, the overall positive effects of employment may become negative when parents are too engaged with work and so less available to their child. An additional explanation may be our relatively crude measure of employment, which simply involved how many parents were in paid employment. The distribution of this variable was highly skewed, with only five children having parents who were both unemployed, limiting the chance of finding significant results. More detailed information related to potential financial strain (i.e. family income), might have produced a different result. Another relevant approach would be to investigate the number of hours worked by each parent, as previous research has found that treatment was particularly effective for children whose parents’ combined number of hours in employment was at least the equivalent of a full time job (Röbl et al. 2013). For this variable, investigating nonlinear relationships may be more informative, as more hours in paid employment might decrease financial strain, but at the same time limit the amount of time a parent can spend with the child on practicing new skills.

Finally, we failed to find the expected moderation effect of family structure. One possible reason for this is that although certain family structures may be more stressful for parents (NSCH 2011/2012), this stress does not necessarily relate to the child’s ASD. Family structure may also be unrelated to the extent to which parents are involved in treatment. A further, methodological explanation is that the power to detect moderation was limited, as only 41 children were not living with two biological parents.

Strengths of this study include its randomized control design, with the RCT protocol being specified before trial initiation (http://www.trialregister.nl, Trial No. 2327) and published prior to completion of the data collection (Hoddenbach et al. 2012). The relatively large sample also allowed for the analysis of parent characteristics potentially related to treatment success. The findings here suggest that parental education levels and parental ASD are important areas for future investigations of moderators of treatment outcome in children with ASD.

Limitations include the absence of detailed diagnostic instruments, such as the Autism Diagnostic Interview-Revised (Lord et al. 1994) or the Autism Diagnostic Observation Scale (Lord et al. 2000), due to limited resources. The inclusion of multiple primary child outcome measures may also be considered a weakness but because of lack of data on the relative sensitivity of any single ToM assessment we chose to use a combination of measures tapping different ToM aspects. This was indicated in the trial protocol (http://www.trialregister.nl, Trial No. 2327), and allowed us to assess whether the training and moderators affected different aspects of ToM. The use of simple categorical measures to assess family structure, parental education/employment, and presence of autism in parents is a further methodological limitation. With respect to the latter variable, for example, a broader measure of autism traits (e.g. Parr et al. 2015a; Pickles et al. 2013) might have produced more meaningful results. Finally, although total sample size was relatively large, some of the subgroups were small, resulting in potential power issues for some moderator analyses. Consequently, findings pertaining to these variables (i.e. parental employment and family structure) should be considered preliminary and require replication in future research. Additional directions for future research include the investigation of other parental variables (e.g. hours parents spent reinforcing the training at home; parenting stress; Osborne et al. 2008), exploration of interactions between different parental variables, and replication in samples with lower verbal IQ scores.

In conclusion, as parents often play an important role in interventions for children with ASD (Burrell and Borrego 2012), investigating which parent characteristics moderate treatment effectiveness is important. The current finding that parental education and parental ASD moderated treatment effects provides valuable information that should be taken into consideration in future treatment design and implementation.
